# Fusion of the genes ataxin 2 like, *ATXN2L*, and Janus kinase 2, *JAK2*, in cutaneous CD4 positive T-cell lymphoma

**DOI:** 10.18632/oncotarget.21790

**Published:** 2017-10-10

**Authors:** Ioannis Panagopoulos, Ludmila Gorunova, Signe Spetalen, Assia Bassarova, Klaus Beiske, Francesca Micci, Sverre Heim

**Affiliations:** ^1^ Section for Cancer Cytogenetics, Institute for Cancer Genetics and Informatics, The Norwegian Radium Hospital, Oslo University Hospital, Oslo, Norway; ^2^ Department of Pathology, The Norwegian Radium Hospital, Oslo University Hospital, Oslo, Norway; ^3^ Faculty of Medicine, University of Oslo, Oslo, Norway

**Keywords:** primary cutaneous CD4 positive T-cell lymphoma, cytogenetics, RNA-sequencing, *ATXN2L*-*JAK2* fusion gene

## Abstract

Acquired mutations were recently described in cutaneous T-cell lymphomas for the *JAK1*, *JAK3*, *STAT3*, and *STAT5B* genes of the JAK-STAT pathway. In the present study, RNA-sequencing of a primary cutaneous CD4 positive T-cell lymphoma carrying a three-way t(9;13;16)(p24;q34;p11) chromosome translocation showed that *JAK2* from chromosome band 9p24 was rearranged and fused to a novel partner gene, *ATXN2L*, from 16p11. RT-PCR together with Sanger sequencing verified the presence of the *ATXN2L-JAK2* fusion transcript. The *ATXN2L-JAK2* fusion gene would code for a chimeric protein containing all domains of ATXN2L and the catalytic domain of the JAK2 tyrosine kinase. The ATXN2L-JAK2 chimeric protein could lead to constitutive activation of the downstream JAK-STAT signaling pathway in a manner similar to that seen for other JAK2 fusion proteins.

## INTRODUCTION

Janus kinase 2 (JAK2), JAK1, JAK3, and Tyrosine kinase 2 (TYK2) constitute the Janus kinase family of intracellular, non-receptor tyrosine kinases [1]. Each protein has 4 different domains. An amino terminal four-point-one, ezrin, radixin, moesin (FERM) domain binds cytokine receptors [2]. The Src Homology 2 (SH2) domain allows the protein to dock to phosphorylated tyrosine residues on other proteins [3]. The pseudokinase domain (JH2) has a critical regulatory function [4], whereas the carboxyl-terminal tyrosine kinase domain (JH1) is catalytic containing typical features of a tyrosine kinase such as conserved tyrosines necessary for JAK activation [5]. Members of the JAK kinase family are involved in the JAK-STAT signaling pathway transducing extracellular cytokine-mediated signals to the nucleus, resulting in expression of a number of genes involved in apoptosis, differentiation, hematopoiesis, immunity, proliferation, and oncogenesis [6–8].

Mutations of the *JAK2* gene and aberrations of chromosome band 9p24, where *JAK2* maps, lead to rearrangements of *JAK2* and have been reported in various hematologic neoplasms [9–11]. The most common point mutation of *JAK2*, V617F, is found in myeloproliferative disorders such as polycythemia vera, essential thrombocythemia, and chronic idiopathic myelofibrosis [9, 11]. It results in constitutive activation of JAK2 which initiates the downstream cascade of the JAK-STAT pathway giving the hematopoietic precursor cells carrying this mutation a proliferative and survival advantage [9–11].

In 1997, Lacronique et al [12] detected an *ETV6-JAK2* fusion gene in a T cell childhood acute lymphoblastic leukemia (ALL) carrying a t(9;12)(p24;p13) chromosome translocation. The same year, Peeters et al [13] reported the same *ETV6-JAK2* fusion and 9;12-translocation in a child with early B-precursor ALL and t(9;12)(p24;p13) and an adult patient with atypical chronic myeloid leukemia with a t(9;15;12) (p24;q15;p13) in the bone marrow cells. A common feature of the ETV6-JAK2 fusion proteins in all three above-mentioned cases was helix-loop-helix (HLH) oligomerization of the ETV6 and JAK2 catalytic domains [12, 13]. In later years, *JAK2* has been shown to be a promiscuous gene forming fusions with 29 different partner genes in hematologic malignancies as well as in solid tumors such as breast carcinoma, kidney carcinoma, lung adenocarcinoma, and squamous cell carcinoma of the oral cavity [14].

In lymphatic malignancies, a t(8;9)(p22;p24) resulting in a *PCM1-JAK2* fusion gene was reported in a patient with peripheral T-cell lymphoma [15], and a t(4;9)(q21;p24) leading to a *SEC31A-JAK2* fusion was found in two patients with classical Hodgkin lymphoma [16]. Both *PCM1-JAK2* and *SEC31A-JAK2* encode constitutively activated tyrosine kinases [15, 16]. It is important to detect and report *JAK2* fusions, regardless of the diagnosis, because such cases could be responsive to treatment with JAK2 inhibitors [17–20].

Primary cutaneous T-cell lymphoma NOS (CTCL) is a heterogeneous group of post-thymic T-cell lymphomas with considerable variation in clinical presentation, including skin manifestations, different histomorphological picture, and immuno-phenotype. They are characterized by different clinical outcomes and treatment considerations [21, 22]. Recently, using next generation sequence methodologies, recurrent mutations were described in the *JAK1*, *JAK3*, *STAT3*, and *STAT5B* genes in CTCL [23–25]. The mutations in *JAK1* and *JAK3* are clustered in the region coding for the pseudokinase domain of the proteins, whereas in *STAT3* and *STAT5B*, the mutations target the portion of the gene encoding the SH2 domain [26]. The mutations lead to an alteration of the JAK-STAT pathway in CTCL.

Here, we present a patient with primary cutaneous CD4+ T-cell lymphoma with t(9;13;16)(p24;q34;p11) as the sole karyotypic aberration. By RNA-sequencing we could demonstrate that a molecular consequence of the translocation was fusion of the genes ataxin 2 like, *ATXN2L*, from 16p11 with Janus kinase 2, *JAK2*, from 9p24.

## RESULTS

### Karyotyping and fluorescence *in situ* hybridization (FISH) analysis

**Figure 1:**
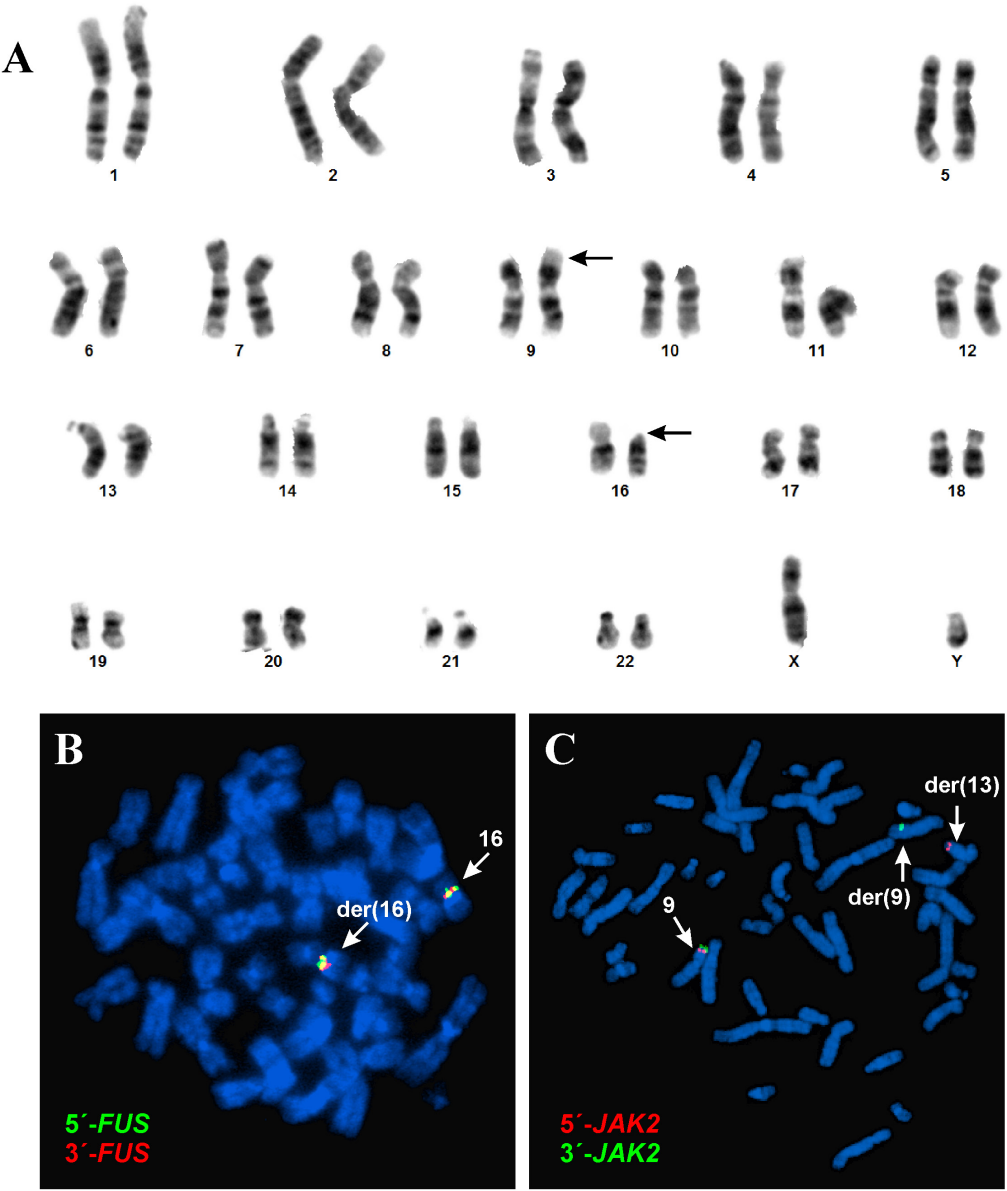
G-banding and FISH analyses of the cutaneous CD4 positive T-cell lymphoma **(A)** Karyotype showing the chromosomal aberrations of neoplastic cells. **(B)** FISH with the *FUS* break apart probe. **(C)** FISH with the *JAK2* break apart probe.

G-banding analysis of short-term cultured lymph node cells yielded the karyotype 46,XY,t(9;16)(p24;p11)[5]/46,XY [8] (Figure 1A).

FISH with a *FUS* break-apart probe showed that the *FUS* gene on 16p11 was not rearranged. Both the green and red probes (corresponding to the 5′- and 3′-end parts of *FUS*, respectively) remained on 16p11 of the der(16)t(9;16) (Figure 1B). FISH with a *JAK2* break-apart probe showed that the *JAK2* gene had been rearranged and that the 5′-end of *JAK2* (red probe in Figure 1C) had moved to the q34 band of a seemingly normal chromosome 13, whereas the 3′-end of the gene (green probe in Figure 1C) remained on the p24 band of der(9)t(9;16). Thus, based on the combined G-banding and FISH analyses, the karyotype was corrected to 46,XY,t(9;13;16)(p24;q34;p11)[5]/46,XY [8].

### RNA-sequencing, molecular genetic analysis

Using the TopHat-Fusion on the raw sequencing data a hybrid sequence was found between chromosome bands 16p11 and 9p24 (Table 1). BLAT of the sequences obtained by TopHat-Fusion on the human genome browser-hg19 assembly (http://genome-euro.ucsc.edu/cgi-bin/hgGateway) showed that the *ATXN2L* gene from 16p11 was fused to the *JAK2* gene from 9p24. These data were confirmed when we used the BLAST algorithm (http://blast.ncbi.nlm.nih.gov/Blast.cgi) to compare the sequences with the *ATXN2L* reference sequence NM_007245 version 3 and the *JAK2* reference sequence NM_004972 version 3. TopHat-Fusion did not detect any fusion sequences between 9p24 and 13q34 nor between 13q34 and 16p11.

**Table 1:** Fusion sequence between chromosome bands 16p11 and 9p24 found using the TopHat-Fusion program on raw RNA-sequencing data

Chromosome	Fusion point	Fifty bases on the left side of the fusion	Fifty bases on the right side of the fusion
16	28847442	ACTCTCAGCTTCCACACCCTCACCCTACCCCTACATCGGACACCCCCAAG	GTGAGCAGCCTGGCCAGGCGCCTGGATTTCCAGGAGGAGCCGATGACAGG
9	5081724	AGAGAATGTTATTTGCTAATTTAAGGTGATAATATTCTTTATTTCTCCAG	ATTATGAACTATTAACAGAAAATGACATGTTACCAAATATGAGGATAGGT

**Figure 2:**
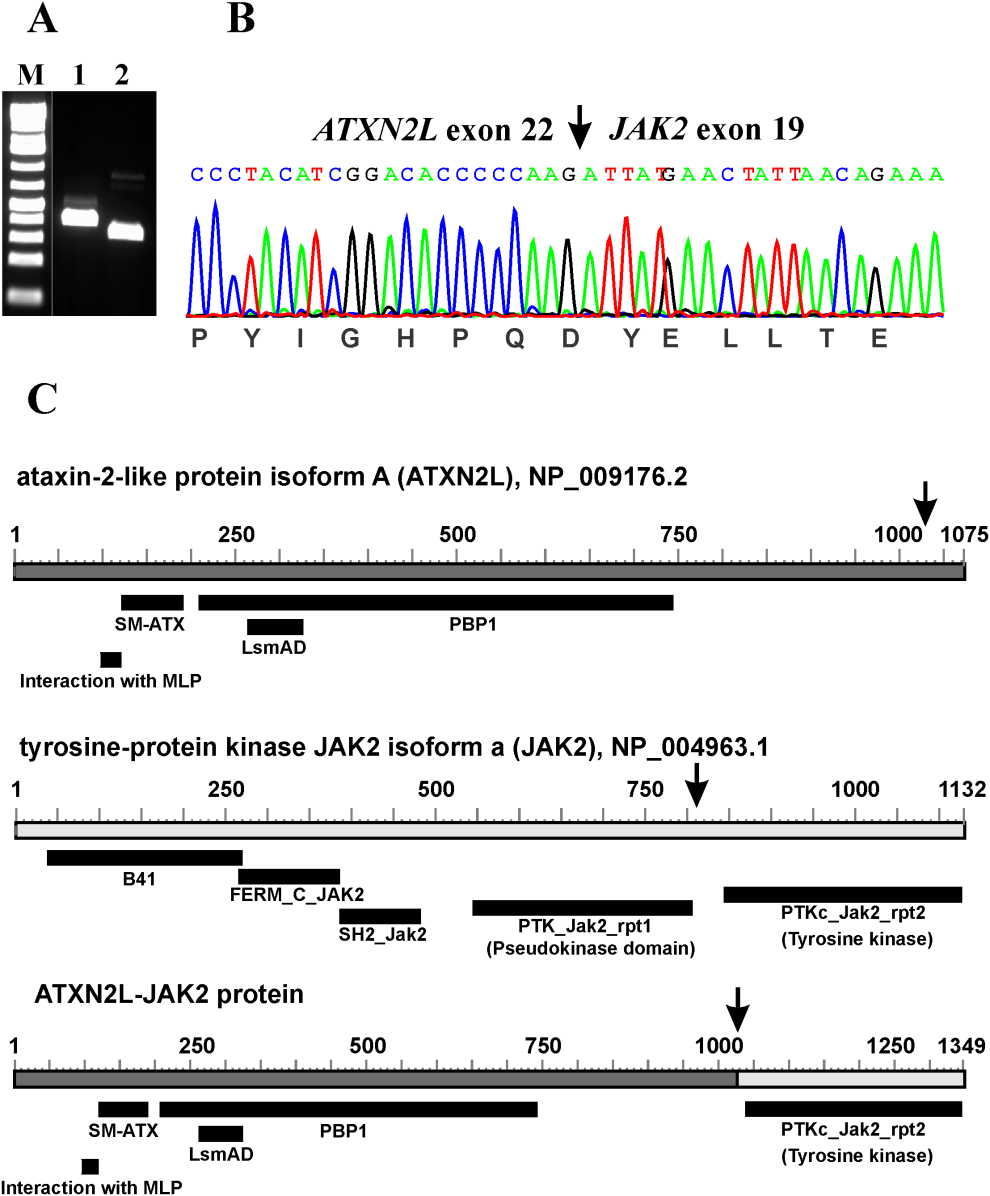
Molecular analyses of the cutaneous CD4 positive T-cell lymphoma **(A)** Nested PCR with the primer combinations ATXN2L-3116F1/JAK2-3006R1 (lane 1) and ATXN2L-3183F1/JAK2-3044R1 (lane 2). M, 1 Kb DNA ladder (GeneRuler, Fermentas). **(B)** Sanger sequencing of the amplified products showed an *ATXN2L-JAK2* cDNA fragment in which the fusion point was identical to that found using TopHat-Fusion. **(C)** Diagrams showing the ataxin-2 like protein isoform A (ATXN2L), NP_009176.2, tyrosine-protein kinase JAK2 isoform a, NP_004963.1, and the putative ATXN2L-JAK2 protein resulting from the fusion between *ATXN2L* (from16p11) and *JAK2* (from 9p24). Arrows indicate breakpoints/fusions.

RT-PCR with the ATXN2L-3108F1 and JAK2-3084R1 primer combination amplified a 358 bp cDNA fragment (data not shown). Nested PCR with the primer combinations ATXN2L-3116F1/JAK2-3006R1 and ATXN2L-3183F1/JAK2-3044R1 amplified 283 bp and 254 bp cDNA fragments, respectively (Figure 2A). Sanger sequencing of the amplified products showed a chimeric *ATXN2L-JAK2* cDNA fragment in which the fusion point was identical to that found using TopHat-Fusion (Figure 2B; Table 1).

## DISCUSSION

In the present study, we showed that the *JAK2* gene was rearranged and fused to a novel partner gene, *ATXN2L*, as a result of a t(9;13;16)(p24;q13;p11) occurring as the sole chromosomal abnormality in a case of primary cutaneous CD4 positive T-cell lymphoma. Karyotyping of G-banded preparations was followed by appropriate FISH and later RNA-sequencing analyses, whereafter the *JAK-ATXN2L* fusion was confirmed using reverse transcriptase PCR and Sanger sequencing.

The *ATXN2L* gene encodes an ataxin type 2 related protein, maps on chromosome band 16p11, 2.4 Mbp distal to *FUS*, and is expressed in several human tissues but particularly in the thymus, lymph nodes, spleen, fetal kidney, and adult testis [27, 28]. The ATXN2L protein is a paralog of ataxin-2 which has been implicated in the neurodegenerative disorder spinocerebellar ataxia type 2 [27–29]. Several alternatively spliced transcripts encoding different isoforms have been found for the gene (https://www.ncbi.nlm.nih.gov/gene/11273). ATXN2L has one region named “interaction with MPL”, an ataxin 2 SM domain (SM-ATX; pfam14438), an LsmAD domain (pfam06741), and the PAB1-binding protein PBP1 which interacts with poly(A)-binding protein (PBP1; COG5180) (Figure 2C). The cancer-relevant function of ATXN2L is unknown, but Meunier et al [28] reported that it interacts with the thrombopoietin receptor MPL, and Kaehler et al [30] found ATXN2L in a complex together with the RNA helicase DDX6, the poly(A)-binding protein, and ataxin-2 indicating that ATXN2L is involved in cellular RNA processing. ATXN2L is a component of stress granules and plays a role in how their formation is regulated [30]. The same group also reported that ATXN2L is asymmetrically dimethylated *in vivo*, that it is associated with arginine-N-methyltransferase 1 (PRMT1), and that the nuclear localization of ATXN2L is altered by methylation inhibition [31].

Based on the karyotyping and FISH data, the *ATXN2L-JAK2* fusion gene should be generated on the der(9) chromosome. Based on the reference sequences NM_007245.3 and NP_009176.2 for *ATXN2L* and NM_004972.3 and NP_004963.1 for *JAK2* (Figure 2C), the *ATXN2L-JAK2* fusion gene should code for a chimeric protein with 1349 amino acid residues. It would contain all domains from ATXN2L and the catalytic domain of the protein tyrosine kinase of JAK2 (Figure 2C). Although no functional studies could be performed to assess the consequences of the present fusion, one can assume that the ATXN2L-JAK2 chimeric protein leads to abnormal phosphorylation of the JAK2 tyrosine kinase and constitutive activation of the downstream JAK-STAT signaling pathway in a similar way to what has been seen with other JAK2 fusion proteins [16].

Studies showing JAK2 aberrations in T-cell lymphomas are limited. However, the *PCM1-JAK2* fusion which was reported in a patient with peripheral T-cell lymphoma [15], the amplification of *JAK2* which was found in 12.5% of cutaneous T-cell lymphomas [23], and the present *ATXN2L-JAK2* fusion gene indicate that *JAK2* is recurrently involved in T-cell lymphomagenesis albeit at as yet unknown frequency. The clinical importance of detecting *JAK2*-fusion genes and proteins is connected with the detection of new targeted molecular therapies that may be effective in such patients [20]. Van Roosbroeck [16] found a *SEC31A-JAK2* fusion in classical Hodgkin lymphoma and showed that SEC31A-JAK2 Ba/F3 transformed cells were sensitive to treatment with JAK2 inhibitors. Classical Hodgkin lymphoma and primary mediastinal large B-cell lymphoma with 9p24.1/*JAK2* copy gain(s) were sensitive to treatment with the JAK2-selective inhibitor fedratinib both *in vitro* and *in vivo* [32]. Additional *in vitro* studies showed that ruxolitinib, an orally administered JAK1/2 selective inhibitor, had significant activity against *PCM1-JAK2*, *ETV6-JAK2*, and *SEC31A-JAK2* fusion genes [16–18]. Ruxolitinib induced cytogenetic as well as clinical remission in patients with t(8;9)(p22;p24)/*PCM1-JAK2*-positive chronic eosinophilic leukemia [17–19, 33]. Schwaab et al [38] reported complete remission on ruxolitinib therapy also in myeloid neoplasms with *PCM1-JAK2* and *BCR-JAK2* fusion genes, albeit of limited duration [34]. Ruxolitinib was shown to be effective in CTCL cases with both JAK1 and JAK3-activating mutations. The drug activates apoptosis and inhibits DNA synthesis [25].

In summary, *JAK* mutations, including *JAK2* fusion genes brought about by cytogenetically visible translocations, may be pathogenetically significant in CTCL, although it is not yet known how widespread their involvement is. Their detection is particularly important since treatment with JAK inhibitors could prove valuable in such cases.

## MATERIALS AND METHODS

### Ethics statement

The study was approved by the Regional Committee for Medical and Health Research Ethics, South-East Norway (REK Sør-Øst; http://helseforskning.etikkom.no) and written informed consent was obtained from the patient to publish the case details. The ethics committee’s approval included a review of the consent procedure. All patient information has been de-identified.

### Case history

A 29-year-old man presented with a 6 month history of a non-itching rash. Clinical examination revealed widespread skin lesions involving the right upper arm, both legs, and the lower part of the abdomen. With the exception of the right upper arm, the other lesions were symmetrically distributed. The rash in the arm area was well demarcated, erythematous, and slightly scaly. Similar changes were seen on the abdomen and legs where also areas of ulceration and vesicles were present. By palpation, enlarged lymph nodes up to 1.5 cm were detected in both the axillary and inguinalregions.

### Morphological findings

**Figure 3:**
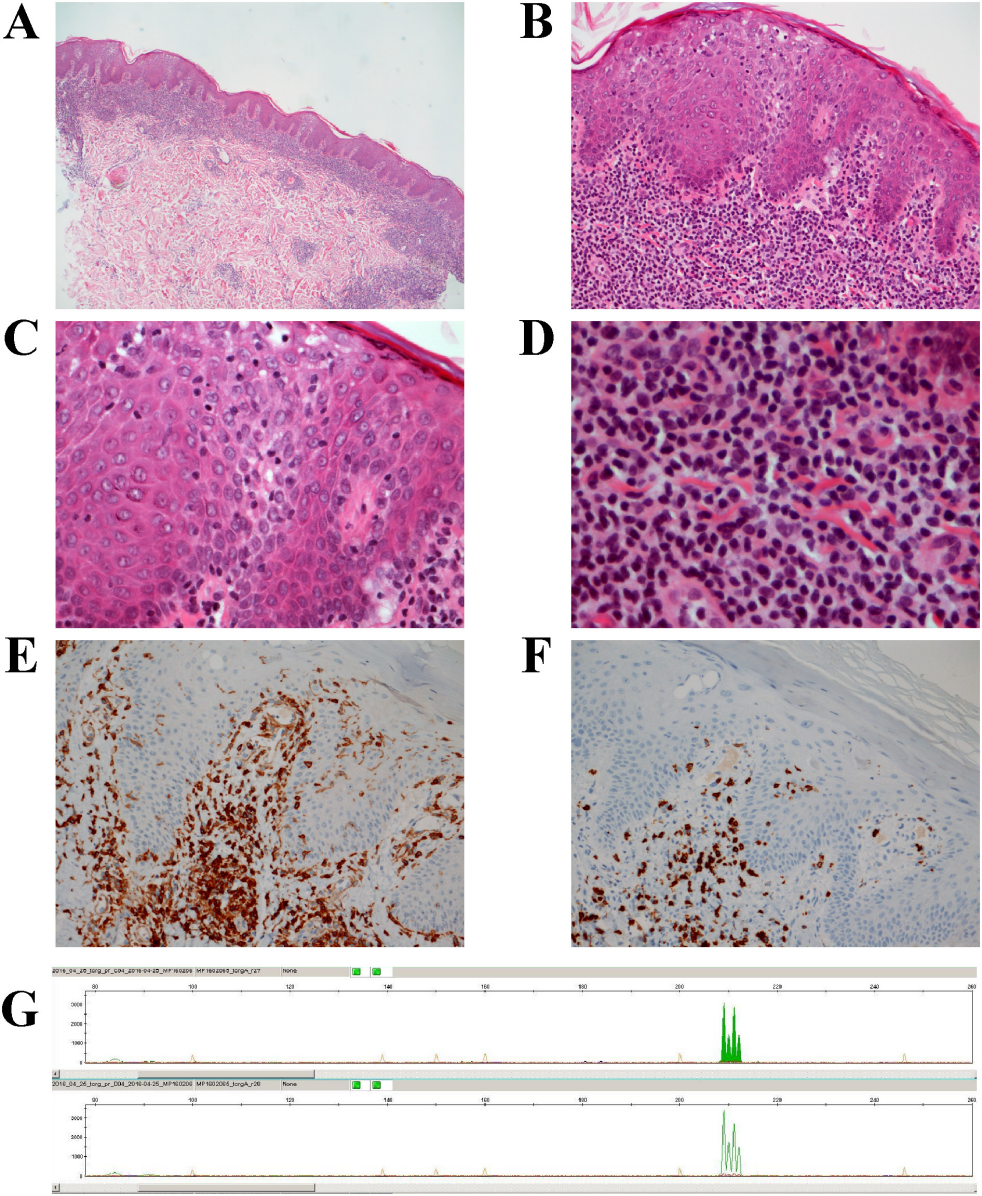
The histomorphological features of the skin lymphoma **(A)** H&E section, magnification x 10: Skin punch biopsy with slightly acantotic epidermis and band-like diffuse lymphoid infiltrate in the papillary and upper reticular dermis. **(B)** H&E section, magnification x 20: Higher magnification showing epidermis with mild spongiosis and a moderate diffuse epidermotropic lymphocytic infiltrate with basal accentuation. **(C)** H&E section, magnification x 40: Higher magnification showing epidermotropic lymphocytic infiltrate with small lymphocytes with small hyperchromatic nuclei. **(D)** H&E section, magnification x 60: Dense, band like infiltrate, composed of small lymphocytes, spread histiocytes, spread pigmented macrophages and single lying Touton type giant cells in the papillary dermis a. **(E)** Immunohistochemical staining with anti-CD4 antibody, magnification x 10. **(F)** Immunohistochemical staining with anti-CD7 antibody, magnification x 10. **(G)** T-cell receptor gamma alpha and beta gene rearrangement showing 4 different picks at 209, 210, 211, 212 bp.

Up to 4 mm skin punch biopsies from the right forearm and left knee were taken and sent for pathological examination. The histological picture was identical in both regions. The epidermis was slightly acantotic with mild spongiosis and focal parakeratosis. Within the epidermis, there was a moderate diffuse epidermotropic lymphocytic infiltrate with basal accentuation (Figure 3A-3C). Pautrier micro abscesses were not detected. In the papillary dermis, a dense, band-like infiltrate was seen composed of small lymphocytes, but with occasional histiocytes, pigmented macrophages, and Touton type giant cells. In the deeper sections, a hair follicle with marked folliculotropic infiltrate of small and middle-sized lymphoid cells was also detected (Figure 3D). There was perivascular and periadnexal infiltration of small lymphocytes in the reticular dermis, again with interspersed single plasma cells and Touton type giant cells. Alcian histochemical staining did not reveal any mucin accumulation.

A septal and centrolobular lymphoid infiltrate with the same cellular composition was seen in the hypodermis.

Immunohistochemical studies showed a mature T-cell immunophenotype with presence of CD4 and loss of CD7 in the dermal compartment and partial loss of CD7 in the epidermal component, mostly in the basal region (Table 2, Figure 3E and 3F). Examination of monoclonal TCR gamma gene detected rearrangements of T-cell receptor gamma, alpha, and beta genes (Figure 3G). Direct immunofluorescence did not show any deposition of immunglobulins (IgA, IgG, IgM), complement, or fibrinogen. New biopsies, taken three months later and after the 5 CHOP cycle, revealed almost the same morphology with a bit less pronounced deep infiltration, but still perivascular and partially diffuse atypical lymphoid infiltration in the upper dermis with scarce epidermotropism. In addition, some epidermal changes attributable to the chemotherapy were also observed. The epidermis showed slight basal degeneration with some spread single keratinocyte necrosis. The immunohistochemical profile and molecular analysis revealed the same immunophenotype and clonal TCR gene rearrangement both epidermis and dermis.

**Table 2:** Results of immunohistochemical markers obtained from epidermal and dermal lymphocytic infiltration

Immunohistochemical markers	Epidermal lymphocytic infiltration	Dermal lymphocytic infiltration
CD2	+/-	+
CD3	+	+
CD4	+	+
CD5	+	+
CD7	+/-	-
CD8	-	-
CD25	-	-
CD30	-	-
CD56	-	-
CD57	-	-
ALK1	-	-
BCL-6	-	-
CXCL-13	-	-
FOXP3	-/+	-
Granzyme B	-	-
TCL1a	-	-
TIA-1	-	-
EBV (ISH)	-	-

### G-banding and karyotyping

Cells from an enlarged axillary lymph node was cultured and harvested using standard techniques [35]. Chromosome preparations were G-banded with Leishman stain and examined. The karyotype was written according to The International System for Human Cytogenomic Nomenclature (ISCN) 2016 guidelines [36].

### FISH analysis

Fluorescence *in situ* hybridization (FISH) was performed on metaphase spreads using the Vysis *FUS* (16p11) break-apart FISH Probe (Abbott Molecular, Illinois, USA) and the Kreatech *JAK2* (9p24) break-apart FISH probe (Leica Biosystems, Newcastle, UK). Fluorescent signals were captured and analyzed using the CytoVision system (Leica Biosystems).

### RNA-sequencing analysis

For extraction of total RNA, the miRNeasy Mini Kit was used (Qiagen Nordic, Oslo, Norway). The RNA quality was evaluated using the Agilent 2100 Bioanalyzer (Agilent Technologies, Santa Clara, CA, USA). Two μg of total RNA were sent for high-throughput RNA-sequencing at The Genomics Core Facility, Oslo University Hospital (http://genomics.no/oslo/). For RNA-sequencing, the Illumina TruSeq Stranded mRNA protocol was used. The software TopHat-Fusion was used for the discovery of fusion transcripts [37, 38].

### Molecular genetic analysis

Two Nested PCR amplifications were used for verification of the fusion product. The primers used for PCR and direct Sanger sequencing are given in Table 3. The procedures of cDNA synthesis, reverse transcriptase-Polymerase Chain Reaction (RT-PCR), and direct sequencing of the PCR products have been described in detail [39]. In the first PCR, the forward primer ATXN2L-3108F1 and the reverse primer JAK2-3084R1 were used. One μL of the first PCR products was used as template in each of the nested PCRs. The primer combinations were ATXN2L-3116F1/JAK2-3006R1 and ATXN2L-3183F1/JAK2-3044R1. For both first and nested PCR amplifications, the cycling conditions were: initial denaturation at 94 °C for 30 sec followed by 35 cycles of 7 sec at 98 °C, 30 sec at 58 °C, and 30 sec at 72 °C, and a final extension for 5 min at 72 °C.

**Table 3:** Primers used for PCR amplification and direct Sanger sequencing analyses

Name	Sequence (5′->3′)	Position	Reference sequence	Gene
ATXN2L-3108F1	GCCCATGTCCAAACTGGAATCA	3108-3129	NM_007245.3	*ATXN2L*
ATXN2L-3116F1	CCAAACTGGAATCACAGCAGCC	3116-3137	NM_007245.3	*ATXN2L*
ATXN2L-3183F1	CTGCACCCACCCCAGAGTCAT	3183-3203	NM_007245.3	*ATXN2L*
JAK2-3084R1	AGAGGGTCATACCGGCACATCTC	3106-3084	NM_004972.3	*JAK2*
JAK2-3044R1	CCCTTGCCAAGTTGCTGTAGAAAT	3067-3044	NM_004972.3	*JAK2*
JAK2-3006R1	TTCAAACTGTGTAGGATCCCGGTC	3029-3006	NM_004972.3	*JAK2*
